# Higher Parity, Pre-Pregnancy BMI and Rate of Gestational Weight Gain Are Associated with Gestational Diabetes Mellitus in Food Insecure Women

**DOI:** 10.3390/ijerph18052694

**Published:** 2021-03-07

**Authors:** Heng Yaw Yong, Zalilah Mohd Shariff, Barakatun Nisak Mohd Yusof, Zulida Rejali, Yvonne Yee Siang Tee, Jacques Bindels, Eline M. van der Beek

**Affiliations:** 1 Department of Nutrition, Faculty of Medicine and Health Sciences, Universiti Putra Malaysia, Selangor 43400, Malaysia; yong_hy@upm.edu.my; 2 Department of Dietetics, Faculty of Medicine and Health Sciences, Universiti Putra Malaysia, Selangor 43400, Malaysia; bnisak@upm.edu.my; 3 Department of Obstetrics and Gynaecology, Faculty of Medicine and Health Sciences, Universiti Putra Malaysia, Selangor 43400, Malaysia; zulida@upm.edu.my; 4 Danone Specialized Nutrition (Malaysia) Sdn. Bhd, Mid Valley City, Lingkaran Syed Putra, Kuala Lumpur 59200, Malaysia; yvonneyeesiang.tee@danone.com; 5 Nutricia Research Foundation, Conradpark 3, 2441 AE Nieuwvee, The Netherlands; jacquesbindels@yahoo.de; 6 Department of Pediatrics, University Medical Centre Groningen, University of Groningen, Hanzeplein 1, 9713 GZ Groningen, The Netherlands; e.m.van.der.beek@umcg.nl

**Keywords:** hemoglobin, hemoglobin change, gestational diabetes mellitus, pregnancy

## Abstract

Food insecurity may exacerbate adverse maternal health outcomes during pregnancy, however, this association has not been well established, particularly in the context of developing countries. This study aimed to identify the associations between household food insecurity and gestational diabetes mellitus (GDM) risk among urban pregnant women. Household food insecurity was assessed using the translated 10-item Radimer/Cornell hunger scale. Logistic regression models were used to estimate the associations between food insecurity status and GDM risk. About 35.6% of women experienced food insecurity, with 25.2% reported household food insecurity, 8.0% individual food insecurity, and 2.4% child hunger. Food insecure women were at significantly higher risk of developing GDM compared to food secure women (AOR = 16.65, 95% CI = 6.17–24.98). The significant association between food insecurity and GDM risk was influenced by pre-pregnancy BMI, parity and rate of GWG at second trimester. Food insecure women with parity ≥ 2 (AOR = 4.21, 95% CI = 1.98–8.92), overweight/obese BMI prior to pregnancy (AOR = 12.11, 95% CI = 6.09–24.10) and excessive rate of GWG in the second trimester (AOR = 9.66, 95% CI = 4.27–21.83) were significantly more likely to develop GDM compared to food secure women. Food insecurity showed strong association with GDM risk in that the association was influenced by maternal biological and physical characteristics. Multipronged interventions may be necessary for food insecure pregnant women who are not only at risk of overweight/obesity prior to pregnancy but also may have excessive gestational weight gain, in order to effectively reduce GDM risk.

## 1. Introduction

Household food insecurity, i.e., the inability to obtain nutritious and safe foods in socially acceptable ways, is increasingly recognized as an independent risk factor for many poor health outcomes among women [1]. Studies have shown that women living in food insecure households had higher prevalence rates of overweight [2,3] and related health complications [4,5,6]. Previous studies reported that food insecurity during pregnancy was associated with greater gestational weight gain (GWG), a greater observed-to-recommended weight gain ratio and increased risk for developing gestational diabetes mellitus (GDM) [7]. Research from both animal and human studies suggest that being worried, concerned or anxious about not having enough food, or uncertainty about one’s ability to obtain food is associated with stress-related weight gain [3,8]. Women from food-insecure households may become economically dependent on low-cost, processed, high-calorie and low-nutrient dense foods in order to stretch the household budget. The consumption of these high energy dense foods over time may lead to excessive weight gain [9].

Among non-pregnant adults, household food insecurity may both predispose to and exacerbate the manifestations of diabetes mellitus [4,5,6]. Household food insecurity was associated with the risk of diabetes after controlling for weight status and other potential confounders [4,5]. Among diabetic adults, household food insecurity was further associated with poor diabetes management [6]. People living in food insecure environment with limited budget often have limited control over their living environments, which can affect their ability to access and prepare healthy food. For example, they are only able to buy foods from low-income neighbourhoods (convenience stores), which carry low-nutrition foods or they may have difficulty to find accommodations with access to a stove and a refrigerator, which are needed to prepare meals, and thus, they often choose easier-to-access food (e.g., canned, pre-cooked) [10,11]. Diets common in food insecure households, such as energy-dense (e.g., refined grains, added sugars, fats), canned or pre-cooked foods may increase dietary glycemia load, and further increase susceptibility to one or more chronic illness, including type 2 diabetes risk [12,13,14].

In Malaysia, the prevalence of overweight and obesity (BMI ≥ 25.0 kg/m^2^) among women aged ≥18 years old increased by 8.7% in the period between 2006 and 2019 [15,16]. With the increasing prevalence of overweight and obesity among women of childbearing age, the risks of having excessive GWG and/or developing GDM are high. A study in rural areas of a state on the east coast of Malaysia showed that about 83.9% of non-pregnant reproductive-age women experienced some forms of food insecurity [17] and hypothesized that food insecurity predisposed these women to excessive weight gain. Entering pregnancy with higher BMI may increase the risk of excessive GWG since pregnancy is a period of adapted physiology and metabolism as well as actual and perceived increase in food intake, thereby increasing the risk of GDM. This study aimed to understand the association between food insecurity and risk of GDM, in particular the specific factors that could influence the association among women living in urban areas. This information is pertinent to the development of public health strategies to prevent GDM in food insecure populations.

## 2. Materials and Methods

**Figure 1:**
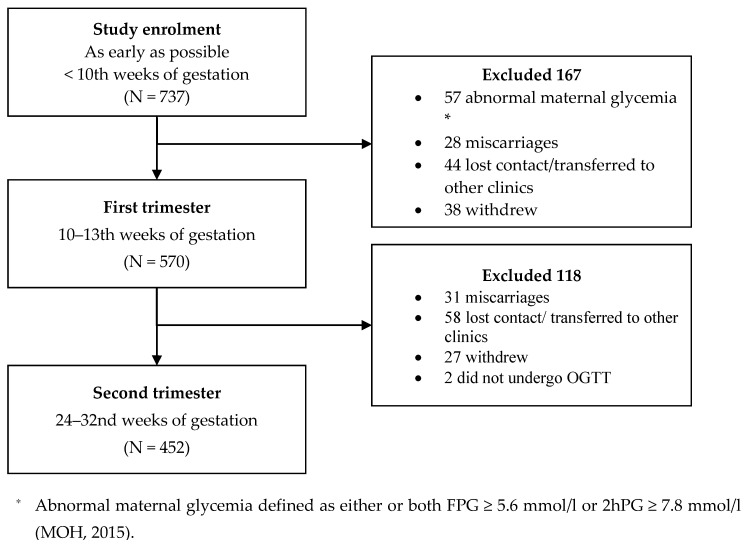
Recruitment of study respondents.

This study used data from the Seremban Cohort Study (SECOST) that examined the determinants and pregnancy outcomes of maternal hyperglycemia. Between January 2013 and December 2013, a total of 737 pregnant women were recruited through maternal and child health (MCH) clinics in Seremban district, Negeri Sembilan. All women were recruited before 14th weeks of gestation and followed through their pregnancy. Women completed face-to-face interviews for information on socio-demographics, dietary intake, physical activity and a retrospective measure of food insecurity status [18]. Of the 737 women enrolled in the study,

The final sample comprised 452 pregnant women (61.3%) who completed follow-up, including the oral glucose tolerance test (OGTT), around 28–32 weeks of gestation. A total of 285 women were excluded due to abnormal maternal glycemia (n = 57) at study enrolment signalling pre-pregnancy type 2 diabetes, miscarriage or stillbirth (n = 59), withdrawal for health/personal reasons (n = 65), transfer to other clinics or lost contact (n = 102), and failure to undergo OGTT (n = 2). (Figure 1).

### 2.1. Food Insecurity

Household food insecurity was assessed between 24 and 32nd weeks of gestation using the 10-item Radimer/Cornell hunger scale [19], which was translated into Bahasa Malaysia [20] (Table 1). Women were asked about the individuals in their households experiencing food insecurity during the woman’s pregnancy. Food insecurity consists of four components: quantity of food, quality of food, food acceptability and certainty of getting food. According to the conceptualization of the Radimer/Cornell Scale, as the problem worsens, household food insecurity representing uncertainty and anxiety about food at the household level is experienced first (mild food insecurity), followed by adult food insecurity (or moderate food insecurity) characterized by decreased quality and quantity of food eaten by adults [21]. Child hunger (or severe food insecurity) is the most severe problem that is characterized by decreased quantity and quality of food eaten by children. Based on the data on the items of the Radimer/Cornell Scale, women were classified into four mutually exclusive categories with increasing severity, e.g., food secure, household food insecure, adult food insecure and child hunger.

### 2.2. Anthropometric Measurements

Maternal height was measured at study enrolment, while weight was measured at enrolment and each study visits using a standard instrument (SECA digital weighing scale and SECA body meter) and standard procedures. Women were requested to recall pre-pregnancy body weight (current pregnancy). Pre-pregnancy BMI was calculated as weight in kilograms divided by the square of height in meters and classified according to World Health Organization (WHO)’s cut-off points respectively: underweight (<18.5 kg/m^2^), normal weight (18.5–24.9 kg/m^2^), overweight (25.0–29.9 kg/m^2^) and obese (≥30.0 kg/m^2^) [22,23]. Weight in the first, and second trimester was measured as the closest measurement to 12th weeks of gestation (10–13th weeks) and to 26 weeks of gestation (range 24–32nd weeks, respectively. Estimates of the GWG at first trimester and rate of GWG at second trimester were then classified as gaining below (inadequate GWG), within (adequate GWG) and above (excessive GWG) the recommendation by the Institute of Medicine (IOM) [24].

### 2.3. Gestational Diabetes Mellitus

All pregnant women were required to take a standard 2-h 75 g oral glucose tolerance test (OGTT) between 28th to 32nd week of gestation [23]. GDM at study period was diagnosed if either or both fasting plasma glucose (FPG) was ≥5.6 mmol/L or 2-h plasma glucose (2hPG) was ≥7.8 mmol/L according to the Ministry of Health (MOH) Malaysia guideline [23].

### 2.4. Other Variables

Socio-demographic information obtained included current age, education level, ethnicity, occupation status, monthly household income, and household size. Obstetrical information (e.g., gravidity and parity) was obtained from medical records.

### 2.5. Statistical Analysis

All statistical analyses were performed using IBM SPSS Statistics for Windows, Version 23.0 [25]. Continuous variables were expressed as the means and standard deviations, while categorical variables as absolute frequencies and percentages. As there were few women in the individual insecure (n = 36) and child hunger (n = 11) categories, the response categories for food insecurity were combined into two groups: food secure and food insecure. Multiple logistic regression was used to determine the association between food security status and the risk of GDM, adjusted for covariates. Further interaction analysis was performed on age, family history of DM, income, parity, rate of GWG and pre-pregnancy BMI. A stratified analysis was performed further for any significant interaction term. Adjusted odds ratios (OR) and 95% confidence intervals (CIs) were calculated. A p-value of 0.05 was set as statistically significant.

## 3. Results

### 3.1. Characteristics of Subjects

Table 2 shows the characteristics of the 452 pregnant women. The mean age of women was 30.3 ± 4.5 years, with 51.3% were aged more than 30 years. Majority of women were Malays (88.9%), had attained secondary and lower education (45.8%), were employed (69.0%), had low household incomes (63.7% reported less than RM 3860/920.69 USD), and had a household containing 3–4 people (50.7%). Most women had no medical history of GDM (93.1%) or family history of diabetes mellitus (75.7%). The mean height, pre-pregnancy weight and pre-pregnancy BMI were 1.56 ± 0.06 m, 58.72 ± 13.36 kg, and 23.96 ± 4.99 kg/m^2^, respectively. Slightly more than half (53.5%) of women were in the normal weight range, 22.4% overweight, 13.5% obese and 10.6% underweight. While most women (90.3%) had inadequate GWG at the first trimester, the proportion of women with inadequate (33.6%), adequate (32.8%) and excessive (33.6%) rate of GWG at the second trimester were equally distributed. Approximately 35.6% of women revealed that they had experienced of food insecurity, with 25.2% (n = 161) reporting household food insecurity, 8.0% (n = 36) individual food insecurity, and 2.4% (n = 11) child hunger. About 10.6% of the women were diagnosed with GDM.

### 3.2. Associations between Household Food Security Status and the Risk of GDM

Table 3 shows the associations between household food security status and the risk of GDM. Women from food insecure households had significantly higher risk of GDM (AOR = 16.65, 95% CI = 6.17–24.98) compared to food secure women. Additionally, there were significant interactions between household food insecurity with parity, pre-pregnancy BMI, and the rate of GWG in the second trimester. Table 4 further shows the AORs and 95% CI for the associations between household food security status and GDM risk stratified by parity, pre-pregnancy BMI and rate of GWG in the second trimester. Food insecure women with parity of two and above (AOR = 4.21, 95% CI = 1.98–8.92), overweight/obesity (AOR = 12.11, 95% CI = 6.09–24.10) and excessive rate of GWG in the second trimester (AOR = 9.66, 95% CI = 4.27–21.83) were at significantly higher risk of GDM compared to food secure women.

## 4. Discussion

The present study showed that food insecure women had significantly higher risk of GDM compared to food secure women. However, the association was specific for women with higher parity, overweight/obesity, and excessive rate of GWG in the second trimester. Previous studies among pregnant women [7,26] and general population [10,11] have also shown that GDM and T2DM were more prevalent in food insecure than food secure households. Although data on household food insecurity and the risk of GDM are scarce [7,26], studies in low income population (e.g., Pakistan, Sri Lanka, Africa, Turkey,) have also reported an association between higher parity, overweight/obesity and excessive GWG with a higher risk of GDM [27,28,29,30,31,32]. As food insecurity is prevalent in low-income population, these findings lend support to food insecurity as an important risk factor for GDM which could be dependent or independent on other risk factors of GDM.

The association between food insecurity and GDM could, at least in part, be explained by unhealthy diets or eating patterns due to limited food budget or income. Food insecure individuals are more prone to consumption of high energy dense foods with low nutritional value [33], and dysregulated eating. The latter is characterized by overconsumption of food when foods are available, and food restriction when food shortages exist [34], as well as emotional eating, which is triggered by emotional distress caused by lack of access to adequate foods [35,36]. The observed unhealthy diets and/or eating patterns could disrupt normal physiology and metabolic regulation and further contribute the development of GDM [20,37]. Previous studies showed that food assistance programs might play a role in dysregulated eating as they are typically disbursed once monthly and could lead to a monthly cycle of feast and famine [38,39]. Studies on recipients of the Supplemental Nutrition Assistance Program (SNAP), the largest food assistance program in the United States, found that recipients were more likely to report days with no eating [40] and had a lower diet quality [41] at the end of the benefit month than non-recipients. As the present study did not assess whether the women received any food assistance programs, it is unknown whether the association between food insecurity and GDM risk is influenced by such programs. A low intake of fruit and vegetables could deprive food insecure individuals of the protective effects of essential nutrients against diseases such as diabetes, which occur as a result of poor nutrition [16,39]. It is also possible that women with food insecurity have limited knowledge of nutrition, and less time and resources to prepare or engage in healthy eating.

It has been suggested that increasing body fat storage is a physiologically regulated response by the body to a threatened food supply, which occurs specifically in women with low socioeconomic status [42,43]. This could explain why food insecure women are predisposed to overweight or obesity at pre-pregnancy, a well-recognized risk factor for excessive GWG and GDM. Further analysis of the present data also showed that the association between food insecurity and excessive GWG is indeed pronounced among overweight/obese women (AOR = 7.76, 95% CI = 2.08–29.02) compared to underweight/normal weight women (AOR = 2.00, 95% CI = 0.52–7.70). The association between overweight/obese food insecure women and higher GWG could be due to unhealthy eating behavior [44], whereby food insecure women tend to choose high-calorie energy dense but low-nutrient foods (lower protein and micronutrients containing foods, but higher intake of energy dense snack foods) [45,46]; and psychological or emotional stress or depression [47]. The association could also be explained by the availability of and accessibility to fast-food chains and the ubiquitous food stalls or hawker foods in Malaysia [48]. In particular, hawker stalls and fast-food chains are associated with foods that are fried or deep-fried, high in sodium and prepared with high-fat containing ingredients e.g., coconut milk [49]. Additionally, the local snacks or delicacies available at food/hawker stalls or at morning and night markets are high in fat and sugar. Such energy dense foods, eaten outside the normal meal moments, may contribute to excessive daily energy intake. Laraia et al. (2013) further showed that food insecure pregnant women with a dietary restraint, defined as the extent to which a person thinks about her diet and weight and tries to restrict dietary intake before pregnancy, had in fact the greatest GWG [44]. Future studies should further investigate the details of eating patterns of women with food insecurity during pregnancy, such as dietary change before and during pregnancy, as well as its association with health outcomes.

More than one-third of pregnant women (35.6%) in this study experienced food insecurity. This finding is consistent with studies conducted in developing countries, such as Iran (30.9–35.1%) [50,51] and Ethiopia (41.0%) [52], but is higher than that of studies conducted in developed countries, in which 2.3–24.0% of pregnant women were found to face food insecurity [7,44]. The difference between studies may be attributed to the tools used to assess food insecurity (e.g., 18-item core food security module, 9-item household food insecurity access scale and 10-item Radimer/Cornell hunger scale), and the characteristics of the study populations (e.g., economic status, education level, and accessibility of food). This finding further reiterates existing evidence that a relatively high proportion of pregnant women in developing countries experience some level of food insecurity during pregnancy. From a health perspective, food insecurity is mainly caused by the problem of food access, whereby having insufficient resources (e.g., lack of purchasing power, lack of physical access) to obtain appropriate foods for a nutritious diet and the problem of food utilization (e.g., diet with adequate energy and nutrients, safe drinking water, proper sanitation etc.) [53]. Thus, effective health programs or interventions, such as nutrition assistance programs, referrals to local food pantries, and nutrition education initiatives, especially among vulnerable groups (e.g., overweight/obese, low-income pregnant women) are needed to ensure optimal maternal and child health.

Several study limitations should be considered. The study findings cannot be generalized to all pregnant women in Malaysia as this study was conducted among pregnant women attending antenatal care at the MCH clinics in Seremban Districts. For example, food insecurity might be overestimated as most of the pregnant women attending MCH clinics (government-funded clinics) are from low to middle income households. The use of the Radimer/Cornell hunger scale to measure food insecurity might not represent the total burden of food insecurity, as this scale did not address several dimensions of food insecurity such as psychological (e.g., stress, depression) and social reasons (e.g., alienation). Additionally, this scale does not capture reasons beyond financial constraints and limited resources [54,55]. The present study did not investigate psychological factors (e.g., stress, anxiety, depression, stressful life events) that could be associated with food insecurity and further leading to emotional eating and GDM. Furthermore, the diagnosis of GDM was based on the local diagnostic criteria. Despite these limitations, this study provides support for the important role of food insecurity in the development of GDM among urban women from predominantly middle- and low-income households in Malaysia.

## 5. Conclusions

Food insecurity is an important risk factor for GDM in this sample of urban Malay women. The association was particularly pronounced among women with parity of 2 or above, overweight/obesity and excessive GWG. While further research is needed to understand the underlying mechanisms driving the effect of food insecurity on GDM, these data do provide opportunities to develop public health and community interventions. Reproductive age women, including pregnant women from food insecure households, should be targeted for specific interventions that address risk factors of diet-related non-communicable diseases. For example, nutrition advice or counselling to increase the use of healthy foods (e.g., accessible, nutrient-dense, diverse types and affordable foods) should be made available not only during pregnancy but also during the postpartum period and prior to subsequent pregnancy.

## Figures and Tables

**Table 1:** The 10 items in the Radimer/Cornell hunger scale.

Level	Items	
Household	1.	I worry whether my food will run out before I get money to buy more.
	2.	The food that I bought just didn’t last, and I didn’t have money to get more.
	3.	I ran out of the foods that I needed to put together a meal and I didn’t have money to get more food.
	4.	We eat the same thing for several days in a row because we only have a few different kinds of food at hand and don’t have money to buy more.
Individual (Adult)	5.	I can’t afford to eat properly.
	6.	I am often hungry, but I don’t eat because I can’t afford enough food.
	7.	I eat less than I think I should because I don’t have enough money for food.
Child	8.	I cannot give my child(ren) a balanced meal because I can’t afford that.
	9.	I know my child(ren) is/are hungry sometimes, but I just can’t afford more food.
	10.	My child(ren) is/are not eating enough because I just can’t afford enough food.

Noted. Food secure: negative answers (never or not true) to all items. Household insecure: positive answers (sometimes true or often true) to one or more items (1–4) but not to adult or child level items. Individual insecure: positive answers to one or more of items (5–8) but not to items (9–10). Child hunger: positive answer to one or more items (9–10).

**Table 2:** Characteristics of women (N = 452).

Characteristics	n (%)	Mean ± SD
Age (years)		30.30 ± 4.50
≤30	220 (48.7)	
>30	232 (51.3)	
Ethnicity		
Malay	402 (88.9)	
Non-Malay	50 (11.1)	
Education level (years)		12.96 ± 2.39
Secondary and lower	207 (45.8)	
STPM/Matric/Diploma/Certificate	148 (32.7)	
Tertiary and above	97 (21.5)	
Occupation status		
Unemployed	140 (31.0)	
Employed	312 (69.0)	
Monthly household income (RM) ^¥^		3705.72 ± 2053.72
Low (<3860)	288 (63.7)	
Middle (3860–8319)	150 (33.2)	
High (≥8320)	14 (3.1)	
Household size		3.77 ± 1.61
≤2	108 (23.9)	
3–4	229 (50.7)	
≥5	115 (25.4)	
Gravidity		2.46 ± 1.48
1	144 (31.9)	
2	127 (28.1)	
≥3	181 (40.0)	
Parity		1.23 ± 1.00
0	163 (36.1)	
1–2	219 (48.5)	
≥3	70 (15.5)	
Medical history of gestational diabetes mellitus (GDM)		
No	421 (93.1)	
Yes	31 (6.9)	
Family history of diabetes mellitus		
No	342 (75.7)	
Yes	110 (24.3)	
Height (m)		1.56 ± 0.06
<1.55	172 (38.0)	
1.55–1.58	131 (29.0)	
≥1.59	149 (33.0)	
Pre-pregnancy weight (kg)		58.72 ± 13.36
Pre-pregnancy BMI (kg/m^2^)		23.96 ± 4.99
Underweight (<18.5)	48 (10.6)	
Normal (18.5–24.9)	242 (53.5)	
Overweight (25.0–29.9)	101 (22.4)	
Obese (≥30.0)	61 (13.5)	
Gestational weight gain (GWG) at first trimester		0.16 ± 0.04
Inadequate	408 (90.3)	
Adequate	44 (9.7)	
Excessive	0 (0.0)	
Rate of GWG at second trimester(kg/week)		0.38 ± 0.12
Inadequate	152 (33.6)	
Adequate	148 (32.8)	
Excessive	152 (33.6)	
Physical activity and dietary intake at the second trimester		
Total physical activity (METs hour/week)		265.34 ± 117.25
Energy (kcal/day)		2172 ± 896.90
Food security		
Food secure	291 (64.4)	
Food insecure	161 (35.6)	
Household insecure	114 (25.2)	
Individual insecure	36 (8.0)	
Child hunger	11 (2.4)	
Maternal glucose level oral glucose tolerance test (OGTT) (mmol/l)		
Gestational age at OGTT performed (weeks)		28.01 ± 0.24
Fasting plasma glucose (FPG)		4.38 ± 0.54
2-h plasma glucose (2hPG)		5.94 ± 1.51
GDM according to MOH criteria ^‡^	48 (10.6)	
GDM according to IADPSG criteria ^§^	57 (12.6)	

^¥^ 10th Malaysia Plan, 1 USD dollar = 4.19 Malaysian Ringgit. ^‡^ GDM according to MOH criteria, either of both FPG ≥ 5.6 mmol/L or 2hPG ≥ 7.8 mmol/L. ^§^ GDM according to International Association of the Diabetes and Pregnancy Study Groups (IADPSG) criteria, either of both FPG ≥ 5.1 mmol/L or 2hPG ≥ 8.5 mmol/L.

**Table 3:** Unadjusted and adjusted odds ratio (OR) and 95% confidence intervals (95% CI) for associations between food security status and gestational diabetes mellitus (GDM) (n = 452).

Variable	GDM (n = 48)
	OR [95% CI]
Food security status ^a^	
Food secure	1.00
Food insecure	16.65 [6.17–24.98] **
Interaction terms	
Food insecure * pre-pregnancy BMI	1.12 [1.08–1.15] **
Food insecure * parity	1.72 [1.33–2.23] **
Food insecure * rate of GWG (2nd trimester)	17.93 [10.35–22.15] **

^a^ Adjusted by age, parity, pre-pregnancy BMI, family history of DM, income, rate of GWG, energy intake and physical activity level. Interaction terms: age, family history of DM, income, parity, rate of GWG, pre-pregnancy BMI, energy intake and physical activity level. Only significant interaction terms showed in the table. * *p* < 0.05, ** *p* < 0.01.

**Table 4:** Adjusted odds ratio (OR) and 95% confidence intervals (95% CI) for associations between household food security and gestational diabetes mellitus (GDM) stratified by parity, pre-pregnancy BMI and rate of GWG (2nd trimester).

	Parity ^a^		Pre-pregnancy BMI ^b^		Rate of GWG (2nd trimester) ^a^	
	<2 (n = 297)	≥2 (n = 155)	UW/NW (n = 290)	OW/OB (n = 162)	Inadequate (n = 152)	Excessive (n = 152)
	Adjusted OR [95% CI]					
Food secure	1.00	1.00	1.00	1.00	1.00	1.00
Food insecure	0.65 [0.08–6.80]	4.21 [1.98–8.92] *	1.58 [0.76–3.30]	12.11 [6.09–24.10] **	0.69 [0.05–9.72]	9.66 [4.27–21.83] **

^a^ Adjusted by age, family history of DM, pre-pregnancy BMI, income, energy intake and physical activity level. ^b^ Adjusted by age, parity, family history of DM, income, energy intake and physical activity level. * p < 0.05, ** *p* < 0.05.

## Data Availability

The data presented in this study are available on request from the corresponding author. The data are not publicly available due to the Medical Research Ethics Committee (MREC), Ministry of Health Malaysia, has imposed on the restriction of disclosure data contain potentially identifying patient information.
